# Identification of Cancer-Associated Circulating Cells in Anal Cancer Patients

**DOI:** 10.3390/cancers12082229

**Published:** 2020-08-10

**Authors:** Thomas J. Carter, Jeyarooban Jeyaneethi, Juhi Kumar, Emmanouil Karteris, Rob Glynne-Jones, Marcia Hall

**Affiliations:** 1 Mount Vernon Cancer Centre, Middlesex HA6 2RN, UK; thomascarter@nhs.net (T.J.C.); rob.glynnejones@nhs.net (R.G.-J.); 2 Department of Life Sciences, Brunel University, London UB83PH, UK; jeyarooban.jeyaneethi@brunel.ac.uk (J.J.); juhi.kumar@brunel.ac.uk (J.K.); Emmanouil.karteris@brunel.ac.uk (E.K.)

**Keywords:** anal cancer, HPV, cancer-associated circulating cells, liquid biopsies

## Abstract

Whilst anal cancer accounts for less than 1% of all new cancer cases, incidence rates have increased by up to 70% in the last 30 years with the majority of cases driven by human papilloma virus (HPV) infection. Standard treatment for localised anal cancer is chemoradiotherapy (CRT). Localised progression is the predominant pattern of relapse but well under 50% of cases are salvaged by surgery, predominantly because confirming recurrence within post-radiation change is very challenging. Identifying cancer-associated circulating cells (CCs) in peripheral blood could offer a corroborative method of monitoring treatment efficacy and identifying relapse early. To study this, nucleated cells were isolated from the blood of patients with anal cancer prior to, during, and after CRT and processed through the Amnis^®^ ImageStream^®^X Mk II Imaging Flow Cytometer, without prior enrichment, using Pan-cytokeratin (PCK), CD45 antibodies and making use of the DNA dye DRAQ5. Analysis was undertaken using IDEAS software to identify those cells that were PCK-positive and DRAQ5-positive as well as CD45-negative; these were designated as CCs. CCs were identified in 7 of 8 patients; range 60–876 cells per mL of blood. This first report of the successful identification of CCs in anal cancer patients raises the possibility that liquid biopsies will find a future role as a prognostic/diagnostic tool in this patient group.

## 1. Introduction

Anal cancer is a rare cancer, accounting for less than 1% of all cancer diagnoses [1] and around 2% of all cancers of the gastrointestinal tract [2]. Squamous cell carcinoma of the anus (SCCA) is the predominant histological subtype, accounting for over 90% [3,4]. Incidence rates of SCCA have increased by up to 70% over the last 30 years, with the majority of cases occurring as a result of human papilloma virus (HPV) infection [5]. Risk factors for SCCA include immunosuppression, multiple sexual partners, history of anal intercourse and smoking [6,7]. Most cases of SCCA are diagnosed at an early stage (T1/T2); metastatic disease accounts for <5% of new cases [8].

The standard treatment for localised SSCA is chemoradiotherapy (CRT) [8]. CRT consists of a pyrimidine analogue (5-fluorouracil or capecitabine) and mitomycin C (MMC) delivered concurrently with radiation treatment [9]. Long-term response rates to this treatment are favorable at 80–90%; however loco-regional failure can occur, especially in patients with T3/T4 disease [10]. Tumour HPV status is an important predictor of response, with HPV-16 or -18 driven SCCA more likely to respond to CRT, whilst HPV-negative SCCA are less likely to respond [8,11]. After definitive CRT, approximately 10–15% of patients have persistent cancer, and 15–30% develop subsequent local recurrence after initial complete response [12]. Cross-sectional imaging (computed tomography; CT or magnetic resonance; MR) is generally used to assess response, although recurrence can be difficult to distinguish from treatment effect in the first 6 months following treatment completion [13]. Assessment prior to this can over-estimate the necessity for salvage surgery, which is successful in fewer than 50% of patients with loco-regional recurrences [10]. Prognosis for patients in whom salvage surgery is unsuccessful and for those with metastatic disease remains poor, with median overall survival (mOS) varying from 8 to 34 months [12,14]. Identification of biomarkers to confirm/refute imaging changes suggestive of recurrent/residual local disease after CRT could identify those who should be offered salvage surgery earlier versus those who could safely be followed-up, thus avoiding unnecessary extensive pelvic surgery. Assessment of circulating cells from liquid biopsies are an emerging technology which could provide valuable information on treatment failure, before recurrence disease is clearly evident via cross-sectional imaging [15].

Liquid tumour biopsies from blood possess two distinct advantages over conventional tumour biopsies; (i) they are minimally invasive and (ii) they can enable the tracking of cancers in real time, including changes in response to treatment [11]. Blood tests able to identify relapse would be invaluable, especially where conventional tissue biopsy is inadvisable due to inaccessible sites of recurrence or where the patient is too unwell [15,16]. Circulating tumour DNA (ctDNA) has begun to transition into the clinic [17,18,19], with potential roles including in the selection of cancer patients with advanced disease for appropriate clinical trials [20]. Studies with digital drop PCR (ddPCR) technology recently utilised to detect HPV16 ctDNA in blood are on-going. To date, HPV ctDNA has been detected in patients with early-stage SCCA, and those with advanced disease, with evidence that HPV ctDNA levels are higher in patients with advanced disease [21,22].

Another avenue interrogating liquid biopsies is to isolate and characterize malignant cells which have detached from the primary tumour mass and entered the blood stream; known as cancer-associated circulating cells (CCs) [23,24]. CCs have been observed in a number of cancers including breast, prostate and colorectal cancer [25], and are normally detected via two methods: CellSearch and ISET (Isolation by Size of Tumor cell) assay. CellSearch uses magnetically tagged antibodies in order to enrich the samples before staining with antibodies which target cell surface markers such as epithelial cell adhesion molecule (EpCAM) to identify malignant cells, and to date, is the only FDA approved method to detect CCs in breast, colorectal and prostate cancer. One major limitation to this method is the use of EpCAM alone to enrich samples and identify CCs. EpCAM is down-regulated in many cancers, especially during the process of metastasis, when epithelial to mesenchymal transition (EMT) occurs [26]. In contrast, ISET uses filtration to enrich the liquid biopsy sample based on size differences of CCs. Filtered samples are then immunostained before observation and identification under a light microscope by a trained user. Although not FDA-approved, ISET can detect non-epithelial CCs using a filtration chamber pressurised at 5–9 kPa to capture cells larger than 8 µm onto a membrane as a method of enrichment. However, there are a high number of false positives detected in volunteer patients using ISET alone, a limitation for any filtration-based method of enrichment [27]. Whilst liquid biopsy technologies are increasingly employed in a number of malignancies, in patients with SCCA, a definitive role has not yet been identified [28]. To the best of our knowledge, there are no studies exploring CCs in anal cancer.

Our group has previously demonstrated the utility of liquid biopsies in order to detect CCs in both ovarian [29] and lung cancers [23]. No prior enrichment is undertaken both to mitigate the loss of CCs during sample processing and to be able to examine all sizes of CCs as it is becoming increasingly recognised that many CCs are smaller than 8 µm. Our group has successfully identified CCs by labelling nucleated circulating cells with pan-cytokeratin (PCK) and CD45 markers. This differentiates epithelial cells (PCK+) from haematopoitic (CD45+) non-epithelial (PCK−) cells within the blood stream. Identification as cancer-associated CCs was further confirmed using WT1 antibody in high-grade serous ovarian cancer patient samples, and TTF1 in lung cancer patient samples [23]. Using these techniques, CCs can be identified and quantified at higher numbers compared to alternative techniques. We have also shown that CC levels fall in response to treatment in ovarian cancer patients [29]. Within the present study, we sought to apply these techniques to peripheral blood samples obtained from SCCA patients before, during and after CRT, to determine whether appropriate PCK+ CCs were present and could be identified. To the best of our knowledge, this is the first study demonstrating the potential presence of cancer-associated non-haemopoietic CCs in this patient group.

## 2. Results

### 2.1. Patient Demographics

Blood samples were collected from eight patients diagnosed with localised SCCA. Patients were staged using the TNM (tumour, nodes, metastasis) staging. All patients had localised disease, the majority were T2 (range T1-4), one patient had local nodal involvement (N1) and no patients had distant metastases (M0). The patients were all female, and they all received radical treatment with chemoradiotherapy (41.4 Gy in 23 fractions with concurrent MMC and capecitabine, as part of the ACT-IV clinical trial to which all eight patients were co-recruited). End of treatment (EOT) MR imaging confirmed a complete radiological response in all patients. To date, no patients have clinical or radiological evidence of disease relapse or recurrence, with three patients (38%) followed up for >12 months from EOT at the time of analysis. In addition, no patients have yet undergone re-biopsy to confirm response due to the risk of post-biopsy necrosis. Baseline blood samples were obtained for all eight patients, with serial blood samples (>3) available for five patients (63%). Full staging and follow-up details can be found in Table 1.

### 2.2. Isolation and Identification of Circulating Tumour Cells

**Figure 1:**
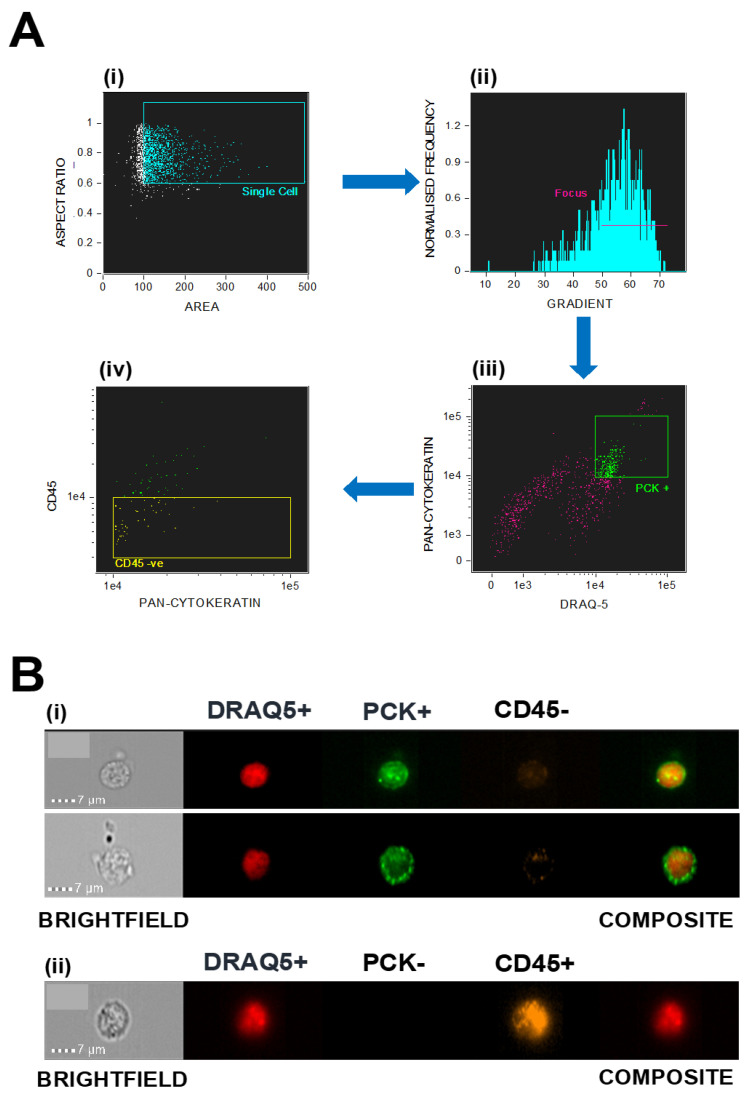
(**A**) Graphs illustrating the criteria used to filter the images taken from ImageStream^®^X Mk II for analysis in IDEAS. Each image is displayed by a single dot and the selection criteria appears either as a box (i, iii, iv) or a line (ii); (i) shows the images filtered *via* size and circularity; (ii) represents the single cells filtered by focus of the images; (iii) shows the in-focus cells that are double-positive stained for PCK and DRAQ5; and (iv) represents the double-positively stained cells that are negative for CD45 staining. (**B**) Images showing (i) two PCK+/CD45− CCs and (ii) an example of a PCK−/CD45+ leucocyte. All images are stained positive with DRAQ5 nuclear staining.

Following sample collection and preparation, cells were stained and processed using the ImageStream^®^X Mk II; images were analysed using the IDEAS software [30]. Collected images were filtered using the criteria as shown in Figure 1A. In brief, images were initially selected based on size and circularity to demonstrate that they are single cells. From this data, only images which were adequately in focus for analytical purposes were chosen. Focused cells that were double-positive for PCK and DRAQ5 were then selected for full assessment and quantification. The final collection of images for every blood sample were then manually analyzed by two independent parties. Positive CCs were identified as being nucleated cells which stained for PCK and DRAQ5 but were negative for CD45. Hematopoietic cells positive for CD45 were excluded from quantification regardless of PCK expression. Examples of CCs and hematopoietic cells identified using IDEAS software in this patient group are shown in Figure 1B. 

### 2.3. Correlation of CC Numbers with Clinical Timepoints

Baseline blood samples were obtained for all eight patients as close to the start of treatment as possible. For four patients, baseline samples were obtained before the first day of CRT, samples for three patients were taken on the first day of CRT, and for one patient, the baseline sample was taken 5 days after starting CRT. With respect to follow-up samples, five patients had a follow-up sample obtained either during CRT or within one week of finishing, five patients had a sample taken between one week and 3 months of finishing CRT and three patients had samples obtained >3 months after finishing CRT (Table 2).

**Figure 2:**
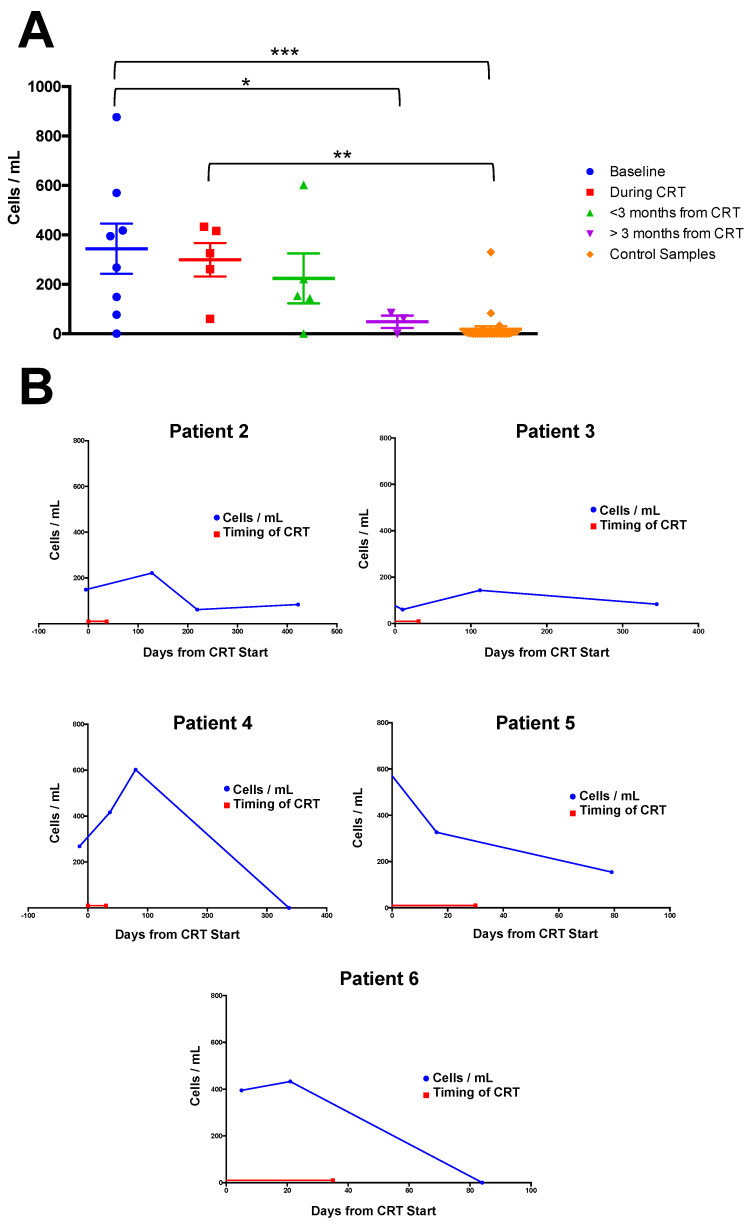
(**A**) CC counts were divided into baseline samples, those taken during CRT (and up to one week after EOT), those taken within 3 months of EOT and those taken over 3 months from EOT. Control samples were obtained from 28 volunteers. When analysed using a one-way analysis of variance (ANOVA), a significant difference was observed between baseline levels and control samples (*p* < 0.001), baseline samples and those taken >3 m from treatment (*p* < 0.05) and between samples taken during treatment and controls samples (*p* < 0.01). (**B**) Individual profiles of CC count (cells/mL) over time in the five patients for whom there were 3 or more samples available. Treatment timing is shown is red, whilst blue lines represent CC counts.

Non-haematopoietic, cancer-associated CCs were identified in baseline samples from seven of eight patients (88%) with a mean number of 334 CCs identified/mL blood (range 0–876). No reason was identified to differentiate the patient in whom no CCs were identified from the remaining seven patients. In control samples, non-hematopoietic CCs were identified in 13 out of 28 volunteers (46%) with a mean of 19 cells/mL blood (0–330). In patient samples taken during CRT (*n* = 5), the mean number of CCs identified was 299/mL blood (60–433), although in four patients, CC numbers increased during CRT. For samples taken within 3 months of completing CRT (*n* = 5), the mean number of CCs was 224/mL blood (0–602), and for those samples obtained >3 months from treatment completion (*n* = 3), the mean number of CCs was 49/mL of blood (0–84) (Figure 2A). For the one patient for whom there were two samples obtained over 3 months from treatment completion (patient 2), only the first sample was included in this analysis.

Following this, patients for whom there were >3 samples (patients 2, 3, 4, 5, and 6) were analysed individually to evaluate changes in CC numbers over time and following treatment (Figure 2B). In one patient (patient 5), CC numbers appear to fall during treatment from baseline, and fell further following treatment. For the remaining four patients (2, 3, 4, 6), CC numbers appeared to increase during, or in the weeks following treatment, with subsequent drops in numbers following this. For patient 2, CC numbers fell to 62 cells/mL >3 months following treatment, but when levels were measured 8 months after this, an increase to 84 cells/mL was observed, the significance of which is unclear. At time of analysis, all patients remained disease-free on clinical surveillance.

## 3. Discussion

We have previously demonstrated the presence of numerous non-hematopoietic CCs in the blood stream of patients with advanced epithelial ovarian cancer and lung cancer, by examining the entire liquid biopsy and avoiding the use of enrichment methods typically employed in CellSearch and ISET technologies [23,29,31,32]. In the present study, we applied these methods to peripheral blood samples obtained from patients with localised SCCA, successfully isolating and identifying cells within the blood of patients which stain positive for the DRAQ5 nuclear marker and the PCK epithelial cell marker, and negative for the pan-hematopoietic maker CD45 for first time (Figure 1). This pattern of expression suggests that these cells represent cancer-associated non-hematopoietic CCs.

Following identification of isolated PCK (+), CD45 (−) CCs, baseline (pre-CRT) CC numbers were compared with samples obtained during CRT, and after CRT and with those from healthy volunteers (Figure 2A). Our results demonstrate significantly higher numbers of CCs in patient samples (7/8 patients (88%), mean of 344 cells/mL, range 0–876, *p* < 0.001), compared to volunteers (13/28 volunteers (46%), mean of 19 cells/mL blood, range 0–330). During treatment, whilst average CC numbers were lower than at baseline, this was statistically non-significant and in four patients, CC numbers actually increased during treatment. However, average CC numbers fell following completion of treatment, with significantly fewer CCs/mL of blood in samples obtained >3 m from the end of treatment (mean 49/mL of blood, range 0–84, *p* < 0.05) compared to baseline; although to date, only 3/8 patients have samples analysed within this time period, and further data collection is ongoing. Taken together, these results suggest a reduction in CC numbers over time from treatment. To provide a clearer impression of the effects of treatment on CC expression, patient samples were then analysed individually (Figure 2B), and this revealed than in 4/5 patients (80%) for whom >3 samples were available, CC numbers appeared to increase during, or in the 3 m following treatment, before subsequently falling. This observation is consistent with previous studies which have shown mobilisation of CCs into the circulation as a result of radiotherapy in patients with lung cancer [33]. Furthermore, an increase in CC numbers in patients during the first three months of treatment for lung cancer was associated with a better progression-free and overall survival [34]. Possible explanations for this include release of CCs prompted by changes in a malignant mass responding to chemotherapy, radiotherapy or damaged during surgical manipulation. Whilst the mechanism is unclear, it is intriguing to consider that the phenotype of the CCs is also important in certain settings. In pancreatic cancer patients, a mesenchymal phenotype for CCs appears to be associated with distant metastases (liver, lung) compared with a pure epithelial CC phenotype which is related to advanced locally recurrent disease [35]. In addition, multicellular, CC microclusters, sometimes including neutrophils, have been identified as a small percentage of the CCs. These microclusters are able to undergo extravasation (via angiopellosis); they have distinct survival and secondary tumour formation abilities, exceeding those of any single CC [36].

During the process of identifying CCs, we too identified various cell doublets including CCs associated with atypical CD45(+), PCK(+) cells (data not shown). The presence of CD45(+), PCK(+) atypical cells associated with CTCs, may represent the presence of atypical cancer-associated macrophage-like cells (CAMLs), which have previously been described in patients with both breast and pancreatic cancer [37]. These CD45(+), PCK(+) cells may either represent macrophages that have engulfed epithelial cellular debris, or CAMLs which have been shown to bind to and migrate in blood attached to CCs [38]. Another possibility is that these cells represent fusions between cancer cells and myeloid cells; atypical cells that may display increased metastatic behavior compared with non-hybrid CCs [39,40]. The presence of these cells demonstrates the complexity of the malignant process and presents an important area of future research to further elucidate the identity of these atypical cells.

Despite these findings, we recognise that there are limitations with the detection of CCs using imaging flow cytometry, as the repertoire of antibody staining is limited for each cell. We have previously demonstrated that using Cell-Free DNA Collection Tubes (Roche), the cell integrity of CCs remains intact for up to 6 days, so we are confident that we are not losing significant numbers of circulating cells or compromising their morphology [29]. We also recognise that CCs may undergo epithelial–mesenchymal transition (EMT) and subsequently loose epithelial markers particularly EpCAM; therefore, it is important in future studies to further characterise the pool of circulating cancer-associated cells using mesenchymal markers such as Vimentin, Fibronectin or N-Cadherin. Furthermore, we and others have also demonstrated the presence of circulating tumor-derived endothelial cells (CTECs) [29], and the properties of endothelial cells within tumours which confer the ability to become CTECs upon entering circulation have recently been the focus of a seminal review article [41]. Further work is on-going to confirm the true prevalence of CTECs in our patient cohorts utilising specific antibodies for endothelial cells (including CD106, CD105, CD34 or CD146). Future work could also exploit the hypoxia driven process of EMT, through the use of emerging technologies to identify abnormal CD31+ CTECS. Identifying these cells in parallel with the PCK+ CCs described in this paper could act as a biomarker not only for treatment response, but also for early metastatic potential [41].

Whilst the results presented showcase convincing data that CCs are present in patients with localised SCCA, further research is needed to fully understand the role of these cells and the implication they have on treatment outcome. After definitive CRT around 10–15% of patients have persistent disease, with a further 15–30% developing subsequent local recurrence after initial complete response. There remains a degree of tension between defining early local recurrence amenable to surgical salvage, and not allowing sufficient time after CRT for a CR to be achieved [12]. Recently, the proportion of patients undergoing salvage surgery seems considerably lower [42] than in older studies [43]**,** reflecting results from the ACT-II clinical trial [12] which demonstrated that delaying surgery is acceptable providing the response trajectory is favourable. Since salvage surgery offers 5-year OS of around 40–45%, serial CC measurements could enable the identification of patients in whom early surgery is necessary versus those who could safely be followed up with imaging; acting as a surrogate marker of response or relapse. Further studies could also investigate whether baseline CC levels are predictive of recurrence. The high levels of CCs in patients undergoing CRT might be indicative of more epithelial cells entering the circulation due to vascular injury. Indeed, increase of CTCs has been documented in patients with advanced esophageal squamous cell carcinoma undergoing CRT [44].

Patient samples continue to be collected for this patient cohort, and future experiments are planned to perform paired ctDNA analysis in an effort to detect and quantify HPV DNA in our patient cohort. Two recent studies have detected HPV ctDNA within the blood of 91.1% of patients with advanced SCCA [45], and 95.6% of patients with early HPV-driven cancers [46]. Combined with knowledge of the immunohistochemical p16 status of our patient cohort, this would provide conclusive evidence that the cells observed and described within this manuscript are cancer-associated CCs. Previously, HPV16 and HPV33 ddPCR assays were used to detect HPV from ctDNA [46]. However, HPV markers can be detected in circulating mononuclear cells from peripheral blood [47]. There is future interest in the application of imaging flow cytometry to our non-enriched cell populations in an attempt to detect HPV-related proteins from liquid biopsies of anal cancer patients.

## 4. Conclusions

In summary, our results confirm the presence of nucleated, non-haematological cells which express epithelial cell surface markers, identified in the bloodstream of patients with clinically localised SCCA. In addition, we have demonstrated that CC numbers fall >3 months following treatment, which is the expected timeframe for response assessment. Data collection is ongoing to evaluate whether CC numbers may provide an early predictor of disease relapse which could be utilised along with serial cross-sectional imaging surveillance to identify those few patients who require radical surgery following CRT for residual or early localised recurrent disease. The identification and quantification of CCs could minimise the mortality among patients with SCCA by providing tailored, personalised, and targeted therapy, whilst also facilitating investigation into new therapeutic targets.

## 5. Materials and Methods

Patient Recruitment: Blood samples were taken from patients enrolled on the CICATRIx clinical study. These patients have advanced cancer and are attending the Mount Vernon Cancer Centre (East and North Hertfordshire NHS Trust). All patients involved in the clinical study provided informed consent for the use of blood samples as well as their participation. The CICATRIx study is a registered and ethics approved study collecting blood samples to explore Circulating tumour cells, cell-free DNA and leucocytes with Imagestream analysis in patients with various cancers. Approved by the West Midlands-South Birmingham Ethics Committee (reference 16/WM/0196).

Blood Sample Preparation: Venepuncture took place using a 21- or 23-G needle, to minimise contamination by skin epithelial cells. Cell-Free DNA Collection Tubes (Roche) were utilized for blood collection to maintain CC membrane integrity for >6 days. A quantity of 1 mL of whole blood from each patient was transferred from Roche tube into a 15 mL Falcon and 9 mL of red blood cell lysis (RBC) buffer was added before gentle rocking and subsequent centrifugation at 2500 rpm for 10 min each to produce a pellet. The process was repeated after removal of supernatant and adding further 3 mL of fresh RBC lysis buffer. Fixation of the pellet was performed by the addition of 1 mL 4% paraformaldehyde in PBS to the pellet and transferring the resultant solution to a 1.5 mL Eppendorf tube. This was left to incubate on ice for 5 min before centrifuging at 4000 rpm for 3 min. After aspiration 1 mL of blocking buffer (10% BSA in PBS) was added and left to incubate on a rotor for an hour followed by addition of antibodies [Pan Cytokeratin (AE1/AE3) and CD45 (Life Technologies)] dissolved in blocking buffer at 1:100 dilution and left to incubate at 4 °C overnight on a rotor. Once the incubation period had elapsed, the sample was centrifuged at 4000 rpm for 3 min and washed with 0.1% Tween in PBS followed by a second centrifugation with the same settings. Finally, 100µL of Accumax cell detachment solution [StemCell Technologies] was added to the pellet alongside 0.5 µL of the nuclear staining DRAQ5 [BioStatus] ready to be run on the Imagestream Mark II [Luminex].

Running and Analysing Samples: The sample was run using the Imagestream Mark II with each staining expressed in a different channel and subsequently, the files produced were analysed and quantified using the IDEAS software [30]. In order to identify a positive CC, the cell had to express positive staining of Pan Cytokeratin and DRAQ5 with a negative staining of CD45 (in order to exclude lymphocytes). All the samples were analysed by two independent observers (JJ and TC). All statistical tests were performed using GraphPad Prism^®^ Software (GraphPad Software). Statistical analyses were performed using one-way ANOVA with significance determined at the level of *p* < 0.05. *p* values are indicated in graphs as follows; * *p* = 0.01–0.05, ** *p* = 0.001–0.009, and *** *p* < 0.0009.

## Figures and Tables

**Table 1:** Patient demographics.

Patient Number	Age at Diagnosis	Tumour Staging	Number of Samples	Imaging Follow Up
1	51	T4N0	1	12 m
2	53	T2N0	4	12 m
3	64	T1N0	4	6 m
4	68	T2N0	4	6 m
5	74	T1/2N0	3	6 m
6	78	T2N0	3	6 m
7	53	T2N1	1	6 m
8	48	T2N0	2	EOT

EOT = End of Treatment (done 3 m following end of CRT), m = months. T = Tumour, N = Nodes.

**Table 2:** CC samples obtained (per patient).

Patient #	Baseline Sample	Sample 2	Sample 3	Sample 4
1	On Day 1	NS	NS	NS
2	Before Day 1	<3 m	>3 m	>3 m
3	On Day 1	During CRT	<3 m	>3 m
4	Before Day 1	During CRT	<3 m	>3 m
5	On Day 1	During CRT	<3 m	NS
6	After Day 1	During CRT	<3 m	NS
7	Before Day 1	NS	NS	NS
8	Before Day 1	During CRT	NS	NS

NS = No Sample.
